# Effects of Different Drying Processes on the Bioactivity and Rutin Content of *Prunu*s spp. (Plums)

**DOI:** 10.1155/2024/9999731

**Published:** 2024-11-16

**Authors:** Eakkaluk Wongwad, Weeraya Preedalikit, Suttida Changprasoed, Suthida Somsai, Nanthawan Singmee, Pawalee Srisuksomwong, Jukkarin Srivilai, Tammanoon Rungsang, Lapatrada Mungmai

**Affiliations:** ^1^Department of Cosmetic Sciences, School of Pharmaceutical Sciences, University of Phayao, Phayao 56000, Thailand; ^2^Research and Innovation Center in Cosmetic Sciences and Natural Products, School of Pharmaceutical Sciences, University of Phayao, Phayao 56000, Thailand; ^3^Division of Science and Mathematics, Faculty of Science and Technology, Phuket Rajabhat University, Phuket 83000, Thailand

**Keywords:** anti-inflammation, antilipid peroxidation, antioxidant, antityrosinase, food, plum extract, rutin

## Abstract

The aim of this study was to investigate the effects of drying process preparations, solvent type, and different species of *Prunus* spp. (European, Japanese, and cherry plums) on bioactive properties (antioxidant, anti-lipid peroxidation, anti-tyrosinase, and anti-inflammatory activities) and rutin content. Leaves and fresh fruits of plums were dried using a cold process (vacuum freeze dryer at −105 ± 5°C) and a hot process (hot air oven at 50 ± 0.5°C). The dried plant material was then extracted using ethanol and propylene glycol to obtain ethanolic crude extracts and propylene glycol extract solutions. These extracts were then tested for phenolic and flavonoid contents and their potential biological activities and analyzed for rutin content using the HPLC method. The results showed that the ethanolic extract of plum leaves obtained via the cold-drying process exhibited higher total phenolic and flavonoid contents (96.94 ± 6.73–112.34 ± 9.08 mg GAE/g sample and 105.10 ± 11.31–185.94 ± 23.35 mg QE/g sample, respectively), as well as greater potential for antioxidant activity (DPPH with IC_50_ = 29.97 ± 1.17 to 31.44 ± 4.16 *μ*g/mL), anti-tyrosinase activity (40.34 ± 1.27%–46.91 ± 0.22%), and anti-lipid peroxidation activity (34.10 ± 4.88%–38.27 ± 2.12%). They also exhibited higher anti-inflammatory activity by inhibiting NO (20.16 ± 0.12%–40.05 ± 0.42%), IL-6 (37.81 ± 3.01%–42.37 ± 8.92%), and TNF-*α* (45.96 ± 7.93%–63.28 ± 6.44%) compared to fresh plum fruit extracts and other extraction procedures. These activities correlated with the rutin content, which was high in the plum leaf extracts (28.52 ± 0.00%–43.82 ± 0.13% *w*/*w*). European plums tended to exhibit greater bioactivities and higher rutin content compared to other species. These findings indicate that plum leaf extracts are a promising source for further applications in food, nutrition, and health products.

## 1. Introduction

The genus *Prunus* is classified within the *Rosaceae* family, which belongs to a group of flowering plants called angiosperms. There are over 35 species of these plants worldwide, native to Asia, Europe, and America [1, 2]. Generally, this plant genus has been grown as an economically valuable crop due to its status as a good source of plum fruit, used in foods, beverages, and health product industries [3]. Plums refer to various shrubs, trees, and their edible fruits in the genus *Prunus*, which are closely related to cherries and peaches. Plum plants can be classified into three main types, including (i) European plum, (ii) Japanese or Chinese plum, and (iii) hybrids of the latter such as cherry plum [4, 5].

Previous research on bioactivity tests of plum extracts indicated that the extract from plum peel has antioxidant, anticancer, and antibacterial activities [6], while the seed extract showed antioxidant and antimicrobial activities [7]. European plum fruit extracts have an inhibiting effect on *α*-glucosidase, *α*-amylase, and pancreatic lipase enzymes [8]. Moreover, these extracts also exhibit hepatoprotective properties [9], along with antioxidant, antibacterial, and anti-inflammatory effects [10]. Leaf extracts of European plum showed larvicidal and repellent activity [11], along with antioxidant, hyaluronidase, elastase, and lipoxygenase inhibitory activities [12], while leaf oil exhibited cholinesterase inhibitory activity [11]. The toxicity study of European plum fruit extracts indicated that the LD_50_ after intraperitoneal injection in mice and rats was more than 3000 mg/kg, and it showed low toxicity when administered orally beyond 15,000 mg/kg [13]. Additionally, acute toxicity in mice and rats of leaf extract is considered safe [14].

The major chemical constituents of plum fruit species, such as European and Japanese plums, have previously been identified, including neochlorogenic acid, quercetin glucoside, rutin, and chlorogenic acid [15, 16]. Additionally, anthocyanidins, such as cyanidin 3-glucoside and peonidin derivatives like peonidin 3-glucoside, are present [15]. Furthermore, chemical constituents of plum leaves, including gallic acid and chlorogenic acid, have also been reported [17]. Based on the various bioactivities of rutin, such as its antioxidant [18–20], anti-inflammatory [21, 22], and anti-tyrosinase [23, 24] properties, it was considered the bioactive marker of the plant in this study.

However, the comparison of the biological activities, plant drying procedures, and extraction methods of the three different known plum species and their plant parts has not yet been established. Various factors contribute to the quality and quantity of plant extracts, including drying techniques, solvent type, extraction methods, and plant species. The influence of drying processes on plant raw materials using different methods has been previously reported and discussed, including techniques such as hot air oven drying, microwave drying, freeze-drying, ultrasound-assisted vacuum drying, and vacuum drying. Different drying techniques result in varying plant yields, phytochemical content, and bioactivity properties [25, 26]. Among these drying techniques, the hot air oven is considered simple and widely used, while freeze-drying is known for preserving active compounds. The polarity of different solvents directly affects the percentage yield and bioactive compounds of the crude extract. Some solvents, such as hexane, chloroform, and dichloromethane, can be toxic, harmful, and damaging to the environment. However, using other organic solvents, such as ethanol (EtOH) and propylene glycol (PG), is considered safer and more environmentally friendly. Depending on the type of plant raw material, the extraction technique plays a crucial role in obtaining the desired extracts. For instance, distillation is used for volatile plant materials, the compression method is used to extract oil from seeds, and maceration is commonly employed for various plant materials.

**Figure 1:**
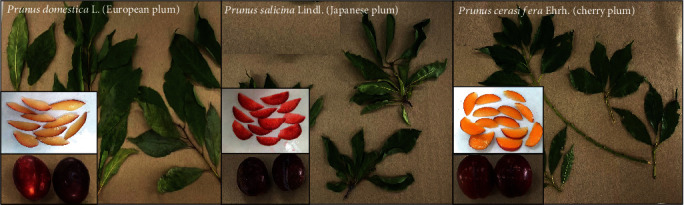
Three varieties of plum leaves and fresh fruits were collected from European plum, Japanese plum, and cherry plum.

In the northern part of Thailand, particularly in Chiang Rai Province, plum species such as *Prunus domestica* L. (European plum), *Prunus salicina* Lindl. (Japanese plum), and *Prunus cerasifera* Ehrh. (cherry plum) (see Figure 1) are extensively cultivated and represent the three main types of plum plants. While the fruits are harvested, the leaves are often considered agricultural waste postharvest. To maximize the economic benefits of plum fruits and the abundant agricultural waste from plum leaves during the harvest period, it is essential to study the preparation processes and bioactivity of these plant materials in greater detail. The objective of this study was to comprehensively explore the effects of drying process preparations, solvent type, and different plum species (European, Japanese, and cherry plums) on bioactive rutin content and bioactivity properties (antioxidant, anti-lipid peroxidation, anti-inflammatory, and anti-tyrosinase activities) for further application and utilization in food, nutrition, and health products.

## 2. Materials and Methods

### 2.1. Chemical and Reagents

The bovine serum albumin (BSA), ferrous sulfate (FeSO_4_), thiobarbituric acid, trichloroacetic acid, dimethyl sulfoxide (DMSO), sodium carbonate (Na_2_CO_3_), phosphoric acid, lipopolysaccharide (LPS), and mushroom tyrosinase were obtained from Sigma-Aldrich (St. Louis, MO, United States). Fetal bovine serum (FBS), Dulbecco's modified Eagle's medium (DMEM), 0.25% trypsin-EDTA, and 10,000 units/mL of penicillin and streptomycin (P/S) were from GIBCO (Grand Island, NE, United States). *L*-DOPA and aluminum chloride hexahydrate (AlCl_3_.6H_2_O) were received from Loba Chemie (Mumbai, India), while the standard compounds, including standard rutin, diclofenac sodium, kojic acid, quercetin, Trolox, and gallic acid, together with macrophage (RAW 264.7) cells and human keratinocyte (HaCaT) cells, were obtained from Sigma-Aldrich (St. Louis, MO, United States). Additionally, the lipid source was purchased from a local market in Mae Tam Market, Muang, Phayao Province, Thailand.

### 2.2. Plant Preparation and Extraction

Leaves and fresh fruits of cherry plum (*P. cerasifera* Ehrh), Japanese plum (*P. salicina* Lindl), and European plum (*P. domestica* L.) were collected from a garden area in Chiang Rai Province, Thailand. The plant specimens were identified by experts from the School of Pharmaceutical Sciences, University of Phayao, Phayao, Thailand. Each voucher specimen of cherry plum, Japanese plum, and European plum was labeled with Lapatrada Mungmai001, Lapatrada Mungmai002, and Lapatrada Mungmai003, respectively, and was retained in the herbarium of the Queen Sirikit Botanic Garden, Chiang Mai Province, Thailand.

The plant samples were prepared using two different methods: plant drying in hot and cold processing. In the hot-drying process, the plant samples were dried at 50 ± 0.5°C for 3 days, while in the cold-drying process, the plant samples were dried in a vacuum freeze dryer (Labconco, United States) (−105 ± 5°C) for 3 days. The plant samples were then ground using a blender, and 50 g of each sample was separately extracted with 200 mL of different solvents: 95% EtOH and PG by maceration for 3 days. Subsequently, to obtain the PG extract solution, the PG extract was filtered, and 3 mg of this extract was weighed and diluted with EtOH to obtain a stock solution of 3 mg/mL. This stock solution was then used for further determination of total phenolic content (TPC) and total flavonoid content (TFC), as well as antioxidant activity assays. To obtain ethanolic crude extract, the ethanolic extract solutions were further evaporated until dryness. The codes for the different extraction process conditions of each sample extract from European, Japanese, and cherry plums are shown in Table 1.

### 2.3. Determination of TPC and TFC

The TPC of the plum sample extracts was analyzed using the previous protocol [27]. One milligram per milliliter of the extract in EtOH was performed. A working concentration ranging from 5.0 to 180 *μ*g/mL of gallic acid was used to create the standard curve.

The TFCs of the plum sample extracts were investigated following the method described earlier [28, 29]. A 0.5 mg/mL solution of the extract in methanol was prepared, and a working concentration ranging from 1.25 to 100.0 *μ*g/mL of quercetin was used to construct the standard curve.

### 2.4. Antioxidant Activity of Plum Extracts Using DPPH Assay

The free radical scavenging activity of 24 extracts of plum leaves and fresh fruits (see Table 1) and standard rutin was evaluated using DPPH assay according to a previously described method with some modifications [30]. In brief, the reaction consisted of 150 *μ*L of DPPH (0.2 mM) in EtOH and 75 *μ*L of various sample concentrations added to a 96-well plate. The mixture was covered with transparent tape to prevent solvent evaporation, shaken to mix well, incubated in the dark for 30 min, and measured at 515 nm using a microplate reader. Trolox was used as the positive control.

### 2.5. Lipid Peroxidation Inhibitory Activity of Plum Extracts Using TBARS Assay

Twelve plum sample extracts, selected for their high antioxidant activity and standard rutin content, were investigated for their ability to inhibit lipid peroxidation using the TBARS assay, following methods from previous studies [29, 31]. Briefly, 20 *μ*L of 2 mg/mL dissolved in EtOH (working concentration) of each sample extract, standard rutin, and Trolox (positive control) was mixed with 140 *μ*L of lipid peroxidation source in a 1.5 mL microcentrifuge tube. This mixture was then incubated at 37°C for 30 min. Subsequently, 20 *μ*L of each 4 mM Fe_2_SO_4_ and 2 mM ascorbic acid was added and incubated for an additional 30 min at the same previous temperature. Following this, 200 *μ*L of TBARS reagent was added and incubated again at 90°C for 60 min. After allowing the test samples to return to room temperature, they were centrifuged at 10,000 rpm and 4°C for 5 min. The supernatant was collected, and 100 *μ*L of it was then measured at 530 nm.

### 2.6. Anti-tyrosinase Activity

Twelve plum sample extracts, selected based on high free radical scavenging activity along with standard rutin, were evaluated for anti-tyrosinase activity following a previously described method [29, 32]. In this process, 1.25 mg/mL in 15% DMSO in 0.6 M phosphate buffer of the test sample was prepared, and 40 *μ*L of the test sample was mixed with 40 *μ*L of 200 U/mL tyrosinase enzyme in a 96-well plate. The volume was adjusted to 160 *μ*L with 80 *μ*L of phosphate buffer and incubated for 10 min at room temperature. Subsequently, 40 *μ*L of *L*-DOPA (2.5 mM) dissolved in phosphate buffer was added, left for 10 min, and then read at 492 nm. Kojic acid at a concentration of 20 *μ*g/mL was used as the positive control.

### 2.7. In Vitro Cell Viability Test

Six plum sample extracts, selected based on higher anti-tyrosinase and lipid peroxidation inhibitory activities and standard rutin, were used for assessment of cell viability in macrophage and human keratinocyte (HaCaT) cells using MTT assay. This method was performed according to a previously described protocol [33]. Both HaCaT and macrophage cells were cultured in DMEM medium supplemented with 10% FBS and 1% P/S solution and incubated in a cell culture incubator at 37°C, 5% CO_2_, and a humidified atmosphere of 95% air conditioning. Upon reaching 80%–90% confluence in 75 cm^2^ cell culture flasks, the cultured cells were trypsinized for further investigation.

To perform in vitro cell viability using the MTT assay, 2.0 × 10^5^ cells/mL of HaCaT cells and 1.0 × 10^5^ cells/mL of macrophage cells were separately seeded in a 96-well plate and incubated in the cell cultured incubator following the previous condition for 24 h. After that, the cells were exposed to 10 serial concentrations (50–1000 *μ*g/mL) of each plum extract. The control cells were treated with 0.002%–1% (*v*/*v*) DMSO in a maintenance medium (used as a blank). Following this, the cells were treated for 24 h. Then, 100 *μ*L of MTT reagent dissolved in growth medium (0.5 mg/mL) was added and incubated for 2 h. After incubation, the growth medium was discarded, and 100 *μ*L of 100% DMSO was added before measuring the absorbance at 550 nm.

### 2.8. Anti-Inflammatory Activities

Six plum sample extracts were tested for anti-inflammatory activities via intracellular NO, proinflammatory cytokines IL-6, and TNF-*α* inhibitory activities.

### 2.9. NO Inhibitory Activity

The plum sample extracts were assessed for NO inhibitory activity following the previous protocol [34]. In brief, 1 × 10^5^ cells/mL of macrophage cells were seeded in a 96-well plate and incubated for 24 h. The cells were then pretreated with 25 *μ*g/mL (final concentration) of each extract, standard rutin, and diclofenac sodium (positive control) for 2 h, followed by 1 *μ*g/mL LPS (final concentration) for 24 h. The supernatant was then collected and assayed as follows: 150 *μ*L of each supernatant solution was mixed with 20 *μ*L of Griess reagent, and the volume was adjusted to 300 *μ*L DI in a 96-well plate and measured at 548 nm.

### 2.10. IL-6 and TNF-*α* Inhibitory Activity

IL-6 and TNF-*α* inhibitory activities of the plum sample extracts and standard rutin were performed using an enzyme-linked immunoassay (ELISA) kit. For this process, 2.0 × 10^5^ cells were seeded in a 24-well plate and incubated for 24 h. After that, the cells were preexposed to 25 *μ*g/mL of each extracted sample, standard rutin, and dexamethasone (positive control) for 2 h, followed by LPS (1 *μ*g/mL) for another 24 h. After incubation, the concentration of IL-6 and TNF-*α* was determined using the ELISA MAXTM Deluxe Set (Biolegend) for human IL-6 and TNF-*α*. The ELISA assay was analyzed and performed following the manufacturer's instructions. The sensitivity of these assays was < 2 pg/mL for IL-6 and 3.5 pg/mL for TNF-*α*.

### 2.11. Determination of Rutin in Plum Leaf and Fresh Fruit Extracts Using HPLC

The amount of rutin content in the extracts of plum leaves and fresh fruits was quantified using the HPLC method. The HPLC instrument consisted of the separation mode (Shimadzu Prominence, Japan) combined with the Shimadzu SPD-10A UV/Vis detector. A reverse-phase C18 column of a KNAUER Vertex III (250 mm × 4.6 mm, 5 *μ*m particle size, Berlin, Germany) was used for the sample separation with an isocratic elution mode (flow rate of 0.8 mL/min) of a mixture of mobile phase (A [1% acetic acid in an aqueous solution] and solvent B [acetonitrile] in a 30:70 ratio). The UV detector was set at 280 nm, the column temperature at 30°C, and the injection volume at 10 *μ*L with a running time of 20 min.

A stock standard rutin solution (1.0 mg/mL in methanol) was prepared and serially diluted to concentrations ranging from 0.008 to 1.0 mg/mL for plotting the calibration curve. In this analysis, 10 *μ*L of a 1 mg/mL solution of each of the six selected plum sample extracts was injected into the HPLC system, analyzed, and calculated.

### 2.12. Statistical Analysis

The experiments were conducted in triplicate (*n* = 3), and all results were expressed as mean values ± standard deviation (SD). Statistical analysis was performed using SPSS Version 24 for Windows.

## 3. Results and Discussion

### 3.1. The Percentage Yields of Plum Extracts

The percentage yields of each plum sample extract are shown in Table 2. The results indicated that the ethanolic crude extract yield of plum leaves, from both hot- and cold-drying processes, ranged from 5.52% to 9.15% (CLHE, ELCE, CLCE, JLHE, JLCE, and ELHE, respectively). Meanwhile, the percentage yield of ethanolic crude extracts from fresh plum fruits increased ranging from 34.85% to 55.82% (JFHE, CFHE, EFHE, CFCE, JFCE, and EFCE, respectively). There was little difference in the percentage yields of leaf extracts between hot- and cold-drying processes (0.05%–2.88%). However, higher yields were observed in the cold-drying process of fresh plum fruit extracts (48.84%, 50.64%, and 55.82% for CFCE, JFCE, and EFCE, respectively) compared to the hot-drying process (34.85%, 35.25%, and 45.75% for JFHE, CFHE, and EFHE), with the difference in yields between the two processes exceeding 10%. Considering the high amount of crude extract yields, this demonstrates that the cold-drying process was more suitable for extraction in both plum leaves and fresh fruit samples than the hot process. Meanwhile, other extractions using PG as a solvent can yield a 100% extract solution. Besides, these propylene extract solutions were maintained in the solution form awaiting further investigation.

### 3.2. TPC and TFC

Phenolic and flavonoid compounds play important roles in numerous biological activities, including anti-inflammatory, antiglycation, antioxidant, and antibacterial properties.  Table 3 presents the results of phenolic and flavonoid content analyses for each plum sample extract. The results indicated that all plum sample extracts contained phenolic compounds ranging from 10.39 ± 1.37 (EFHP) to 112.34 ± 9.08 (JLCE) mg GAE/g extract. TPC values were higher in plum leaf extracts, ranging from 21.43 ± 1.39 to 112.34 ± 9.08 mg GAE/g extract, compared to fresh plum fruit extracts, which ranged from 10.39 ± 1.37 to 24.75 ± 2.78 mg GAE/g extract. Furthermore, based on the extraction solvent used, ethanolic crude extracts exhibited higher TPC values (15.43 ± 2.11 to 112.34 ± 9.08 mg GAE/g extract) compared to PG extract solutions (10.39 ± 1.37 to 32.77 ± 2.06 mg GAE/g extract). TPC results indicated that all plum sample extracts contained phenolic compounds, which was consistent with previous reports on plum plants [35–37].

The results of the TFC analysis for all plum sample extracts showed a similar pattern to that of TPC, with higher levels of flavonoid compounds found in the plum leaf extracts than in the plum fresh fruit extracts. The TFC in ethanolic crude plum leaf extracts ranged from 98.01 ± 5.03 (ELHE) to 185.94 ± 23.35 (JLCE) mg QE/g extract, while in ethanolic crude extracts of fresh fruits, it was 3.14 ± 0.39 (JFCE) to 8.10 ± 0.22 (EFCE) mg QE/g extract. Furthermore, lower TFC were found in PG plum leaf and fresh fruit extract solutions, with values ranging from 0.12 ± 0.02 (JFHP) to 11.38 ± 0.67 (ELCP) mg QE/g sample. TFC results corresponded with previous reports indicating that flavonoid compounds were found in plum extracts [38, 39]. Additionally, identified flavonoids obtained in plum extract, including quercetin, rutin, quercetin glycosides, apigenin, kaempferol, kaempferol glycosides, and catechin, have been reported earlier [40].

The influence of the drying process on the plum samples also affected the levels of TPC and TFC in the sample extracts. The cold-drying process at a low temperature (−105 ± 5°C) using a vacuum freeze dryer for 3 days resulted in a tendency for higher TPC and TFC levels compared to the hot-drying process (50 ± 0.5°C) for 3 days (see results in Table 3). This is because high temperatures might degrade phenolic and flavonoid compounds in the sample extracts, leading to lower TPC and TFC in each sample extract [41]. The fact that high temperatures affect TPC and TFC levels was confirmed by a previous report on European plums, which indicated that a high drying temperature at 60°C led to lower phenolic and flavonoid compound content in sample extracts compared to a drying process at −80°C [26]. Additionally, variations in TPC and TFC levels were observed among different plum species, including European, Japanese, and cherry plums.

### 3.3. Antioxidant Activity

Twenty-four extracts of plum leaves and fresh fruits were tested for their ability to be antioxidant via the hydrogen transfer mechanism using the DPPH method. The results showed that all the sample extracts exhibited antioxidant activity via the hydrogen transfer mechanism, with the IC_50_ values classified into three groups: low, moderate, and high activity. In our study, a high IC_50_ value was classified as ranging from 0.01 to 0.10 mg/mL, moderate activity fell within the range of > 0.10 to 0.50 mg/mL, while values above 0.50 mg/mL were labeled as low activity (Table 3). High antioxidant activity was observed in both hot- and cold-dried ethanolic extracts of plum leaves, with IC_50_ values ranging from 29.97 ± 1.27 to 38.95 ± 0.66 *μ*g/mL (CLCE, ELCE, CLHE, JLCE, ELHE, and JLHE). Among these, CLCE exhibited the highest activity, while JLHE showed the lowest. Meanwhile, moderate antioxidant activity was found in the fruit extracts, particularly in CFCE, EFCE, JFCE, CFCP, and EFCP, with IC_50_ values ranging from 164.83 ± 20.67 to 488.60 ± 30.00 *μ*g/mL. Low antioxidant activity was observed in ELCP, CFHE, CLCP, JFHE, JFCP, ELHP, EFHE, CFHP, JLCP, JLHP, CLHP, JFHP, and EFHP, with IC_50_ values ranging from 622.03 ± 53.38 to 3093.67 ± 462.26 *μ*g/mL. Within this group, ELCP exhibited the highest activity, while EFHP showed the lowest.

The standard rutin exhibited high antioxidant activity with an IC_50_ of 12.55 ± 1.07 *μ*g/mL, while Trolox (a positive control) exhibited an IC_50_ of 4.70 ± 0.33 *μ*g/mL, respectively. These results indicated that the leaf part demonstrated higher antioxidant activity than the fresh fruit part, which can be attributed to the higher levels of phenolic and flavonoid contents found in the leaf part compared to the fresh fruit part of the plant. Based on the solvent used, the ethanolic crude extracts of plum leaves showed an estimated > 20-fold higher antioxidant activity than the PG extract solution. The antioxidant of the ethanolic crude extract of fresh fruit was also higher than that of the PG extract solution. Furthermore, a higher temperature (50 ± 0.5°C) during the drying process affected the lower antioxidant activity of the plum extracts compared to the cold drying process (−105 ± 5°C) that was observed. These results demonstrated that some active compounds, especially phenolic compounds, were decomposed during the hot preparation process, leading to a decrease in antioxidant activity. In addition, the variety of plum species was found to slightly-moderately affect antioxidant activity.

The antioxidant activity of plum extracts has also been noted previously [16, 42, 43]. The potent antioxidant activity of the extracts might stem from standard rutin, an active compound of the plum plant, which showed strong free radical scavenging in our experiment, as well as various reports of its high antioxidant activity [18–20]. Other active compounds corresponding to antioxidant activity obtained from plum extracts, such as gallic acid, chlorogenic acid, neochlorogenic acid, syringic acid, anthocyanins (cyanidin 3-glucoside and cyanidin 3-standard rutinoside), and peonidin derivatives, have been noted [15, 16]. Indeed, based on the consistently high levels of phenolic and flavonoid contents, as well as antioxidant activity of plum extracts, from both hot- and cold-prepared processes of plum leaves and fresh fruits using EtOH, EFHE, EFCE, JFHE, JFCE, CFHE, CFCE, ELHE, ELCE, CLHE, CLCE, JLHE, and JLCE were chosen for further investigation.

### 3.4. Lipid Peroxidation Inhibitory Activity

Lipid peroxidation is an important process that occurs naturally in the human body when free radicals attack lipids, resulting in peroxide or hydroperoxide derivatives. This phenomenon leads to lipid degradation and cell damage, contributing to various pathologies such as aging and inflammation.

**Figure 2:**
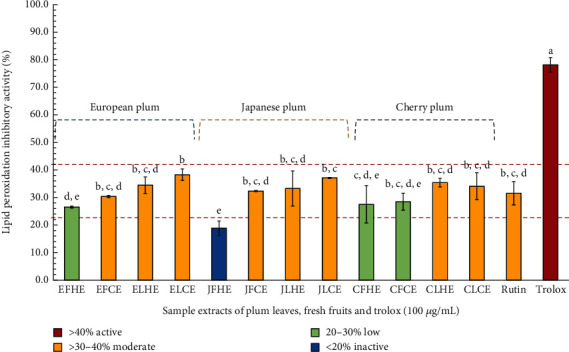
Lipid peroxidation inhibitory activity of each plum leaf and fresh fruit extract and standard rutin (100 *μ*g/mL) using TBARS assay. Trolox (100 *μ*g/mL) was used as positive control. Data are presented as mean ± SD (*n* = 3). Groups that share the same letters (a–e) do not exhibit significant differences at *p* > 0.05 (Tukey's HSD test). Samples inhibiting < 20% were considered as inactive, inhibiting 20%–30% were low activity, inhibiting > 30%–40% were moderate activity, and inhibiting > 40% were demonstrated as active.

The results indicated that the plum sample extracts (100 *μ*g/mL) exhibited moderated activity, with percentage inhibitory activity ranging from 30.38 ± 0.54% to 38.27 ± 2.12% (see Figure 2). Moderate activity was observed in EFCE, JFCE, JLHE, CLCE, ELHE, CLHE, JLCE, and ELCE, with the lowest and highest activity within this group found in EFCE and ELCE, respectively. However, some extracts exhibited low activity, including EFHE, CFHE, and CFCE, with inhibition percentages of 26.54 ± 0.38%, 27.49 ± 6.78%, and 28.46 ± 3.08%, respectively. Meanwhile, JFHE was considered inactive, showing 18.90 ± 2.59% inhibition. The standard rutin at 100 *μ*g/mL demonstrated moderate activity with 31.54 ± 4.23% inhibition, while Trolox (the positive control) at 100 *μ*g/mL exhibited high activity with 78.17 ± 2.59% inhibition. These classifications of inhibitory activity levels followed the previous description by Saesong et al. [31].

The inhibition pattern was similar to antioxidant activity, with the plum leaf extracts showing higher activity compared to plum fresh fruit extracts. However, moderate activity was also observed in some plum fresh fruit extracts, such as EFCE (30.38 ± 0.38%) and JFCE (32.34 ± 0.21%). These results demonstrated variations among plum species and different parts of the plant in terms of lipid peroxidation inhibitory activity. Notably, the ethanolic crude extract of European plum leaves processed by the cold-drying process exhibited the highest activity, with 38.27 ± 2.12%. Previously reported studies on the *P. domestica* L. (European plum) seed extract [44] and fruit of *P. pandus* in inhibiting lipid peroxidation have also been noted [45]. However, it can be noted that the inhibition of lipid peroxidation in Japanese and cherry plums was reported for the first time.

Based on our results, the lipid peroxidation inhibitory activity of the plum leaf extracts might be attributed to other active compounds in the extracts as well as standard rutin, which exhibited moderate activity in this study. Additionally, a previous study on the ability of standard rutin to inhibit lipid peroxidation had been reported. In that study, standard rutin demonstrated high activity, with 68.8% inhibition observed at 0.5 mg/mL. A decrease in lipid peroxidation in human sperm was observed with 30 *μ*M of standard rutin [46], and standard rutin can prevent lipid peroxide products through oral administration in isoproterenol-induced rats [47]. In addition, other active compounds considered bioactive compounds of plum species that play an important role in lipid peroxidation inhibitory activity, such as chlorogenic acid [48], gallic acid [49], and cyanidin 3-glucoside [50], have also been published.

### 3.5. Anti-tyrosinase Activity

The oxidation of *L*-DOPA, resulting in the pigmentation of skin known as melanin, is a natural process that occurs in human skin affected by the tyrosinase enzyme. This enzyme serves as a precursor to the reaction and is involved in melanin biosynthesis. To prevent or slow down this process, inhibiting tyrosinase would be advisable, as it can decrease melanin production, leading to skin lightening.

**Figure 3:**
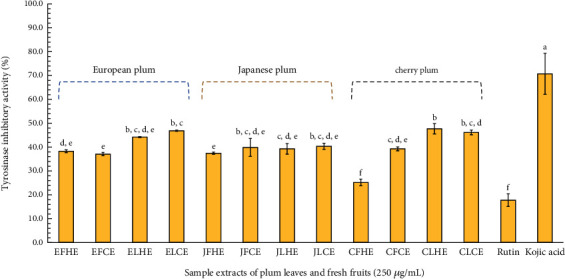
Tyrosinase inhibitory activity of each plum leaf and fresh fruit extracts and standard rutin (250 *μ*g/mL) using anti-tyrosinase assay. Kojic acid (20 *μ*g/mL) was used as positive control. The data are presented as mean ± SD (*n* = 3). Groups that share the same letters (a–f) do not exhibit significant differences at *p* > 0.05 (Tukey's HSD test).

In this study, 12 selected plum sample extracts were tested for their tyrosinase inhibitory activity, and the results are expressed in Figure 3. Each sample extract (250 *μg*/mL, final concentration) showed inhibition of tyrosinase activity ranging from 25.23 ± 1.38% to 47.70 ± 2.17%. Plum leaf extracts exhibited higher inhibitory activity (39.29 ± 2.20%, 40.34 ± 1.27%, 44.22 ± 0.20%, 46.19 ± 0.99%, 46.91 ± 0.23%, and 47.70 ± 2.17% for JLHE, JLCE, ELHE, CLCE, ELCE, and CLHE, respectively) compared to fresh plum fruit extracts (25.23 ± 1.38%, 37.11 ± 0.63%, 37.45 ± 0.46%, 38.31 ± 0.59%, 39.29 ± 0.79%, and 39.88 ± 3.75% for CFHE, EFCE, JFHE, EFHE, CFCE, and JFCE, respectively). Among the leaf extracts, higher inhibitory activity was observed in CLHE, ELCE, CLCE, and ELHE, while the lowest activity was found in CFHE. Meanwhile, standard rutin (250 *μ*g/mL) and kojic acid (20 *μ*g/mL) exhibited inhibition rates of 17.82 ± 2.64% and 77.80 ± 0.04%, respectively. The higher activity in the plum leaf extracts was associated with their greater phenolic and flavonoid content. Additionally, a tendency toward increased tyrosinase inhibitory activity was observed in extracts prepared using the cold-drying process compared to those prepared using the hot-drying process across all tested plum species. The anti-tyrosinase activity of plum extract has been studied earlier in guinea pigs-UVB-induced skin, and the results showed that it inhibited tyrosinase enzyme expression more than the control (> 65%) [51]. Furthermore, the leaf extract of European plum was also observed to have anti-tyrosinase activity with 11.6% inhibition [12].

In this study, the anti-tyrosinase activity might arise from other active compounds obtained from the plum extracts as well as standard rutin, which was present in higher amounts in the plum leaf extract compared to the fresh fruit extract. Previous studies indicated that standard rutin exhibited anti-tyrosinase activity with an IC_50_ of 57.98 *μ*M [23], and supercritical water (SCW)–treated standard rutin at a concentration of 1 mg/mL showed tyrosinase inhibitory activity of 60.05% [24]. On the other hand, other active compounds might exert a synergistic effect on tyrosinase inhibitory activity, such as gallic acid (IC_50_ = 3.59 *μ*M) and quercetin (IC_50_ = 5.26 *μ*M) [52, 53]. The varying potential of phenolic and flavonoid compounds in anti-tyrosinase activity is related to the position and number of hydroxyl groups on the aromatic ring and the presence of sugar moieties in the molecular structure, as noted in previous studies [53, 54].

In addition, based on higher activity in antioxidant, anti-lipid peroxidation, and anti-tyrosinase activity in each part of the sample extracts, six plum extracts, including plum fresh fruit extracts (EFCE, JFCE, and CFCE) and plum leaf extracts (ELCE, JLCE, and CLCE), were chosen for further investigation in anti-inflammatory activity.

### 3.6. In Vitro Cell Viability Test

To obtain safety dosage information for six selected plum extracts (EFCE, JFCE, CFCE, ELCE, JLCE, and CLCE), chosen based on their high activity in inhibiting lipid peroxidation and tyrosinase as well as antioxidant activity, we conducted MTT assay tests on macrophage cells. These tests were aimed at determining the safety dosage of the extract samples for further investigation of their anti-inflammatory activity by inhibiting NO, IL-6, and TNF-*α* factors. Additionally, we also evaluated the effects of the plum sample extracts on HaCaT cells.

**Figure 4:**
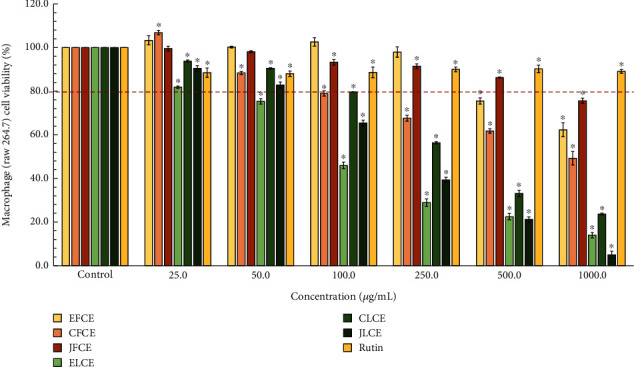
Effects of each plum leaf and fresh fruit extract, as well as standard rutin, on macrophage cell viability, were assessed using the MTT assay. The data are presented as mean ± SD (*n* = 3) of the percentage of cell viability of cells treated with extracts and standard rutin at various concentrations (25–1000 *μ*g/mL) for 24 h. ∗ indicates significant differences compared to the control at *p* < 0.05 using Student's *t*-test.

**Figure 5:**
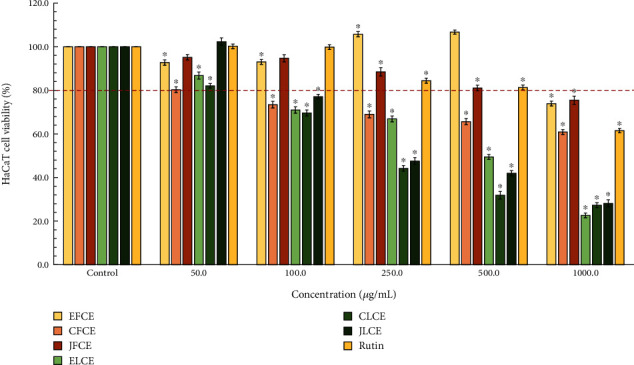
Effects of each plum leaf and fresh fruit extracts as well as standard rutin on HaCaT cell viability were evaluated using the MTT assay. The data are presented as mean ± SD (*n* =3) of the percentage of cell viability of cells treated with extracts and standard rutin at various concentrations (50–1000 *μ*g/mL) for 24 h. ∗ indicates significant differences compared to the control at *p* < 0.05 using Student's *t*-test.

The results of the effect of plum sample extracts on the cell viability tests on macrophage and HaCaT cells are depicted in Figures 4 and 5, respectively. The macrophage cell viability tests for each plum sample extract indicated a trend of decreasing viability with increasing concentration (25–1000 *μ*g/mL) of the extract. Notably, the JFCE sample demonstrated the highest safety profile, with cell viability maintained above 80% until the concentration reached 500 *μ*g/mL. However, viability slightly decreased when the concentration rose to 1000 *μ*g/mL, reaching approximately 75%. The cell survival pattern closely resembled that of the EFCE sample. However, the CFCE showed higher toxicity compared to other plum fresh fruit extracts. Furthermore, CFCE (250 *μ*g/mL) and EFCE (500 *μ*g/mL) caused a decrease in cell viability to below 80%, with approximately 70% and 77% viability observed for CFCE and EFCE, respectively. The ELCE exhibited the most significant effect on the cells, with viability dropping below 80% at 50 *μ*g/mL and reaching approximately 17% viability at 1000 *μ*g/mL. On the other hand, the CLCE and JLCE samples also affected cell viability, with viability below 60% at 250 *μ*g/mL, 40% at 500 *μ*g/mL, and 30% at 1000 *μ*g/mL. Additionally, the effect of standard rutin on macrophage cell viability showed a high safety profile, with cell viability maintained above 80% throughout the tested concentrations.

Considering the effect of each sample extract on HaCaT cell viability, it was found that decreased cell viability was higher in plum leaf extracts (ELCE, CLCE, and JLCE) than that of plum fresh fruit extracts (EFCE, CFCE, and JFCE) (see Figure 5). The toxicity patterns of the sample extracts were similar to those observed in HaCaT cells. JFCE and EFCE exhibited a safer profile compared to others, while ELCE and JLCE showed higher toxicity to the cells. Additionally, standard rutin maintained higher than 80% cell viability across all concentrations ranging from 50 to 500 *μ*g/mL; however, at 1000 *μ*g/mL, cell viability dropped below 80%. These results could be discussed to show that plum fresh fruit extracts are safer than plum leaf extracts. The harm to the cell viability of plum leaf extracts might be attributed to contenting higher phenolic and flavonoid concentrations and other chemical constituents than fresh fruit extract which might affect the macrophage cell viability. Excessive high levels of phenolic compounds affected the cell viability of the V79 cell line was also noted [55].

In conclusion, based on the results, the optimal concentration of all sample extracts and standard rutin was determined to be 25 *μ*g/mL. At this concentration, macrophage cell viability exceeding 80% was observed, prompting its selection for further investigation of NO, IL-6, and TNF-*α* inhibitory activities.

### 3.7. Anti-Inflammatory Activities

#### 3.7.1. NO Inhibitory Activity

NO is a colorless, odorless gas and small lipid-permeable free radical molecule produced by nitric oxide synthase (NOS), including inducible, neuronal, and endothelial NOS isoforms. These signaling molecules are involved in the inflammatory process. However, excessive NO production levels can result in various free radicals directly affecting cells, leading to cell damage and death. Moreover, it can contribute to several physiological and pathological processes, including inflammation and vasodilation. Thus, inhibiting excessive NO production or decreasing the NO level could prevent cell death and the inflammatory process.

**Figure 6:**
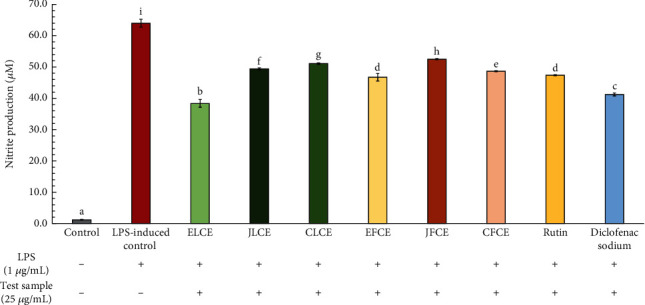
Inhibitory effects on nitric oxide production by the extracts and standard rutin in LPS-induced (1 *μ*g/mL) macrophage cells. Diclofenac sodium (25 *μ*g/mL) was used as the positive control. The data are presented as mean ± SD (*n* = 3). Groups that share the same letters (a–i) do not differ significantly from each other at *p* > 0.05 (Tukey's HSD test).

In this study, macrophage cells were induced with LPS to stimulate NO production, and the supernatant was then tested using the Griess reaction colorimetric assay kit to assess the effect of each plum sample extract on NO inhibition. The results are shown in Figure 6, indicating that after cells were induced with LPS, they could secrete NO, as determined by NO_2_ levels, up to 64.00 ± 0.07 *μ*M (LPS-induced control group), compared to 1.18 ± 0.13 *μ*M in the control group (non-LPS-induced). The level of NO_2_ decreased when treated with sample extracts. This decrease was observed in all sample extracts, as well as in standard rutin and diclofenac sodium (positive control). ELCE exhibited the highest NO inhibitory activity (40.05 ± 0.42%), with a significant reduction (*p* < 0.05) from 64.00 ± 0.07 to 38.37 ± 0.27 *μ*M, compared to the LPS-induced control group. Potent inhibitory activity was also observed with diclofenac sodium (35.58 ± 0.77%, 41.23 ± 0.49 *μ*M). Meanwhile, the NO inhibitory activity of other sample extracts (EFCE, CFCE, JFCE, CLCE, and JLCE) ranged from 17.92 ± 0.31% to 26.99 ± 0.31% (52.52 ± 0.20 to 46.73 ± 0.20 *μ*M), while standard rutin exhibited 25.97 ± 0.23% inhibition (47.38 ± 0.15 *μ*M). These results demonstrated that both plum leaf and fresh fruit extracts possess NO inhibitory properties with similar effects, except for the notably higher activity in the ELCE sample. Additionally, European plum leaf and fresh fruit extracts (ELCE and EFCE) showed a greater tendency for NO inhibitory activity compared to other species.

The NO inhibitory activity of plum extracts from our experiment was correlated to the earlier studies by Najafabad and Jamei [38]. They reported that ethanolic fresh and dried European plum extracts exhibited NO inhibition with values of 8.51 ± 1.09% and 76.02 ± 2.15%, respectively. This activity was also supported by the findings of Ullah et al. [8], which indicated that European fruit plum extract can inhibit NO with an IC_50_ of 0.46 mg/mL.

The NO inhibitory property of the plum sample extract might stem from the effect of standard rutin, which exhibited moderate activity in this test. However, other phenolics and flavonoids, which contained high amounts in the extracts (see Table 3), might be responsible for this activity. Their antioxidant capacity enables them to compete with oxygen in combination with NO, thus reducing nitrite radical formation and leading to the production of the reducing form of NO [56]. Furthermore, other active compounds such as quercetin and gallic acid may play an important role in this activity. As previously mentioned, quercetin, at concentrations of 10 and 30 *μ*M, can suppress NO production by approximately 31% [57] and 56% [58], respectively. Additionally, the suppression of eNOS degradation, leading to a decrease in the level of NO, has also been noted for gallic acid [59].

#### 3.7.2. IL-6 and TNF-*α* Inhibitory Activities

The inflammatory process is one of the body's immune system's key responses, classified into acute and chronic inflammation. Typically, cellular damage activation and the presence of both noninfectious and infectious agents trigger inflammatory signaling pathways through NF-*κ*B, JAK-STAT, and MAPK pathways. This leads to the secretion of various essential proinflammatory cytokine mediators such as IL-1, IL-6, and TNF-*α*, as well as prostaglandins like PGE_2_, PGD_2_, and PGI_2_. However, in this research, the plum sample extracts were assessed for anti-inflammatory activities by inhibiting IL-6 and TNF-*α* after inducing macrophage cells with LPS.

**Figure 7:**
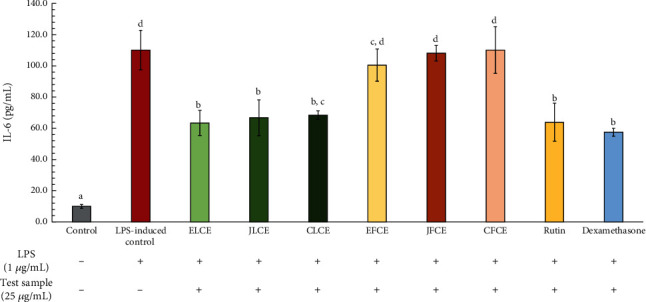
Inhibitory effects on IL-6 production by the extracts and standard rutin in LPS-induced (1 *μ*g/mL) macrophage cells. Dexamethasone (25 *μ*g/mL) was used as the positive control. The data are presented as mean ± SD (*n* = 3). Groups that share the same letters (a–d) do not differ significantly from each other at *p* > 0.05 (Tukey's HSD test).

The effect of plum sample extracts and standard rutin on IL-6 inhibitory activity is illustrated in Figure 7. This result indicates that after inducing macrophage cells with LPS, the IL-6 production increased from 9.99 ± 1.23 to 110.10 ± 12.65 pg/mL. Subsequently, the IL-6 production decreased in treated cells with plum sample extracts and standard rutin. Higher inhibition of IL-6 production was observed in ELCE, JLCE, CLCE, and standard rutin, with IL-6 inhibition rates of 42.37 ± 8.92% (63.45 ± 9.82 pg/mL), 39.33 ± 12.71% (66.80 ± 14.00 pg/mL), 37.81 ± 3.01% (68.47 ± 3.32 pg/mL), and 41.99 ± 13.58% (63.87 ± 14.95 pg/mL), respectively. These IL-6 inhibitory activities were statistically significant (*p* < 0.05) compared to other samples and controls. Additionally, dexamethasone, the positive control, exhibited a high potency in reducing IL-6 production with a value of 57.51 ± 3.09 pg/mL, corresponding to 47.77 ± 2.81% inhibition.

In contrast, the plum fresh fruit extracts (CFCE, JFCE, and EFCE) showed a similar trend in reducing IL-6 but with weaker activity, with inhibition rates of 3.42 ± 1.05% (106.33 ± 1.15 pg/mL), 6.45 ± 0.91% (103.00 ± 1.00 pg/mL), and 12.01 ± 1.09% (96.87 ± 1.20 pg/mL), respectively. However, these reductions were not significantly different compared to the LPS-induced control. Based on our results, it could be indicated that the plum leaf extracts in all species showed more potency in IL-6 inhibitory activity than the plum fresh fruit extracts.

**Figure 8:**
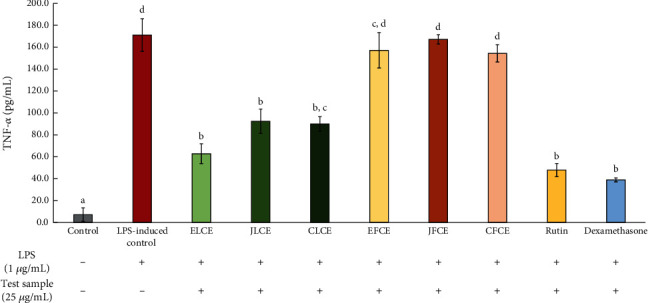
Inhibitory effects on TNF-*α* production by the extracts and standard rutin in LPS-induced (1 *μ*g/mL) macrophage cells. Dexamethasone (25 *μ*g/mL) was used as the positive control. The data are presented as mean ± SD (*n* = 3). Groups that share the same letters (a–d) do not differ significantly from each other at *p* > 0.05 (Tukey's HSD test).

The tendency of TNF-*α* inhibitory activity of the plum sample extracts and standard rutin was similar to that of IL-6 inhibitory activity, with plum leaf extracts showing higher potency in inhibiting TNF-*α* compared to plum fresh fruit extracts (see Figure 8). When macrophage cells were induced by LPS, the proinflammatory cytokine TNF-*α* was secreted at a higher level, increasing to 171.08 ± 14.82 pg/mL from 7.25 ± 6.18 pg/mL in the control group. After treatment with the plum sample extracts and standard rutin, high activity was observed in standard rutin and ELCE, with approximately 72.03 ± 4.30% and 63.28 ± 6.44% reduction in TNF-*α* levels (47.85 ± 7.35 and 62.81 ± 11.02 pg/mL, respectively). Furthermore, high inhibitory activity was also observed in CLCE and JLCE, with TNF-*α* levels at 89.98 ± 6.75 (47.41 ± 4.83%) and 92.45 ± 11.08 pg/mL (45.96 ± 7.93%), respectively, while the TNF-*α* level with dexamethasone treatment was 38.99 ± 1.76 pg/mL or 77.21 ± 1.26% inhibition. These inhibitory activities were significantly different (*p* < 0.05) compared to the LPS-induced control. Additionally, all plum fresh fruit extracts (JFCE, EFCE, and CFCE) exhibited low activity (2.19 ± 1.49%, 7.87 ± 2.24%, and 2.19 ± 1.49%, respectively) in this assay. However, the reduction of TNF-*α* levels in these group extracts was not significantly different (*p* > 0.05) compared to the LPS-induced control.

The high potency of IL-6 and TNF-*α* inhibitory activities might stem from the standard rutin, which exhibited strong IL-6 and TNF-*α* inhibitory effects. These results correlate with a previous report indicating that only 10 *μ*M of rutin had a significant inhibitory effect on both IL-6 and TNF-*α* [21]. Another explanatory report noted that the anti-inflammatory activity of rutin involves the inhibition of IL-6 and TNF-*α*, along with the blocking of the NF-*κ*B activation pathway in LPS-induced mouse muscle cells (C2C12) [22]. This information helps confirm that the major active compound playing an important role in the anti-inflammatory properties of the extracts might be standard rutin.

Furthermore, certain plum species, such as Japanese plum, have been noted previously for their anti-inflammatory properties by inhibiting TNF-*α*, IL-1*β*, and IL-18 in monosodium urate-induced macrophage cells [60]. Additionally, European plum has also been noted for its anti-inflammatory activity by suppressing cyclooxygenase-2 (COX-2) expression in LPS-induced macrophage cells [61], while the suppression of COX-2, TNF, and IL-1*β* was also investigated in cherry plum [62].

### 3.8. Determination of Rutin in Selected Six Plum Extracts Using HPLC Method

**Figure 9:**
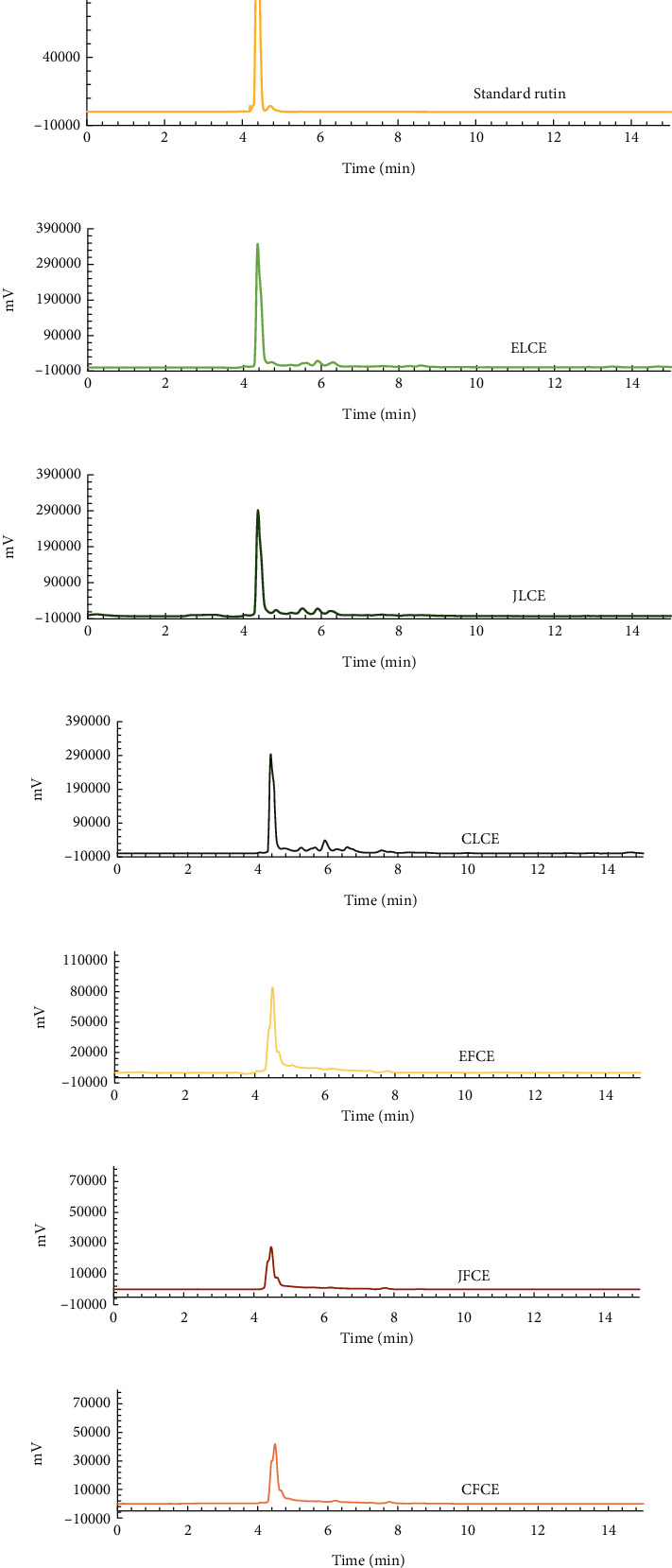
HPLC chromatograms of (a) reference standard rutin (125.0 *μ*g/mL), (b) ELCE, (c) JLCE, (d) CLCE, (e) EFCE, (f) JFCE, and (g) CFCE at a concentration of 1000.0 *μ*g/mL detected at 280 nm.

Six potential plum sample extracts (EFCE, JFCE, CFCE, ELCE, JLCE, and CLCE) were selected to determine the rutin constituent using the HPLC method. The level of rutin in each extract was calculated using the regression equation of the standard rutin calibration curve (*Y* = 9103.9*x* + 60755; *R*^2^ = 0.9999). The HPLC chromatograms of each extract and standard rutin are shown in Figure 9. The quantitative analysis of rutin in each extract is shown in Table 4.

The results indicated that higher amounts of rutin were found in all leaf extracts: JLCE, CLCE, and ELCE, with values of 28.52 ± 0.00, 37.39 ± 0.07, and 43.82 ± 0.13% *w*/*w*, respectively. Lower amounts were found in plum fresh fruit extracts, ranging from 3.39 ± 0.02% to 12.85 ± 0.08% *w*/*w*. Additionally, statistical analysis of the amount of rutin obtained in each plum sample extract showed that all extracts were significantly different (*p* < 0.05) from each other, and the plum leaf extracts are a good source of rutin compound, especially the ELCE sample from European plum species.

These results confirmed that the high amount of rutin in plum leaf extracts corresponds to higher bioactivity, including antioxidant, anti-tyrosinase, anti-lipid peroxidation, and anti-inflammatory activities (IL-6 and TNF-*α* inhibition) compared to plum fresh fruit extracts. Variations of plum species affected by the rutin content are observed.

On the other hand, rutin and other active compounds obtained from European plum leaf extract, such as rutin, quercetin, resveratrol, kaempferol, syringic acid, myricetin, o-coumaric acid, gallic acid, and benzoic acid, with concentrations of 1.359 ± 51.33, 3.839 ± 113.00, 4.060 ± 152.00, 2.678 ± 75.00, 3.300 ± 80.00, 3.180 ± 21.00, 6.836 ± 570.01, 2.991 ± 225.00, and 24 ± 1.41 *μ*g/g leaf, respectively, have been reported [42]. Furthermore, active compounds in European plum fruit extract, including a combination of neochlorogenic acid and its *cis*-neochlorogenic acid, chlorogenic acid, p-coumaroyl quinic acid, and cryptochlorogenic acid, with amounts of 575, 94, 32, and 22 mg/kg, respectively, have also been determined [16]. Additionally, quercetin 3-galactoside (3.5 ± 0.4 mg/100 g fresh weight), quercetin 3-glucoside (1.2 ± 0.1–2.2 ± 0.3 mg/100 g fresh weight), cyanidin 3-glucoside (1.9 ± 0.8–13.5 ± 2.0 mg/100 g fresh weight), cyanidin 3-rutinoside (14.1 ± 1.6–33.0 ± 2.0 mg/100 g fresh weight), peonidin 3-glucoside (1.1 ± 0.2–1.2 ± 0.2 mg/100 g fresh weight), and rutin (2.80 ± 7.7 ± 1.3 mg/100 g fresh weight) have been noted in fresh plum fruit [15].

## 4. Conclusion

The effects of drying process preparation, solvent type, and the different species of plum (European, Japanese, and cherry plums) on their phytochemical composition, bioactive rutin content, and bioactivity properties (including antioxidant, anti-tyrosinase, anti-lipid peroxidation, and anti-inflammatory activities) were successfully explored.

The ethanolic crude extracts of plum leaves, prepared using a cold-drying process with a vacuum freeze dryer at −105 ± 5°C for 3 days, yielded crude extracts ranging from 6.27% to 8.23% *w*/*w* for CLCE, ELCE, and JLCE. These extracts demonstrated higher phenolic content (96.94 ± 6.73–112.34 ± 9.08 mg GAE/g sample) and flavonoid content (105.10 ± 11.31–185.94 ± 23.35 mg QE/g sample), along with enhanced antioxidant (IC_50_ = 29.97 ± 1.17 to 31.44 ± 4.16 *μ*g/mL), anti-tyrosinase (40.34 ± 1.27%–46.91 ± 0.22%), and lipid peroxidation inhibitory activity (34.10 ± 4.88%–38.27 ± 2.12%). Additionally, they showed higher anti-inflammatory activity, as evidenced by inhibition of NO (20.16 ± 0.12%–40.05 ± 0.42%), IL-6 (37.81 ± 3.01%–42.37 ± 8.92%), and TNF-*α* (45.96 ± 7.93%–63.28 ± 6.44%) compared to fresh plum fruit extracts and other extraction procedures.

The bioactivity of the plum extracts correlated with their rutin content, which was high in the plum leaf extracts (28.52 ± 0.00%–43.82 ± 0.13% *w*/*w*) and demonstrated significant potential in various bioactivities, particularly antioxidant (IC_50_ = 12.55 ± 1.07 *μ*g/mL) and anti-inflammatory activities (inhibition of IL-6 and TNF-*α* at 41.99 ± 13.58% and 72.03 ± 4.30%, respectively). The European plum species consistently exhibited higher rutin content and greater bioactivity, especially in anti-inflammatory effects, compared to other species.

These findings demonstrate that ethanolic plum leaf extracts prepared using a cold-drying process are a promising source of antioxidant, anti-lipid peroxidation, anti-tyrosinase, and anti-inflammatory properties. However, this study is limited by the small sample size and the restricted variety of cultivated plum species that might affect the chemical and bioactivity profiles of the plum plant as well as the determination of other active compounds in the extract. Further investigations into the underlying mechanisms of plum leaf extract bioactivity at the molecular level, as well as the quantification of other active compounds and evaluations of efficacy and safety in clinical studies, are essential for the utilization of the extract in food, nutrition, and health products.

## Figures and Tables

**Table 1:** Codes for the different extraction process conditions of European, Japanese, and cherry plum leaves and fresh fruits.

**Plum species**	**Part of plant**	**Drying process**	**Solvent**	**Code**
*Prunus domestica* L. (European plum, E)	Fresh fruit	Hot	EtOH	EFHE
			PG	EFHP
		Cold	EtOH	EFCE
			PG	EFCP
	Leaf	Hot	EtOH	ELHE
			PG	ELHP
		Cold	EtOH	ELCE
			PG	ELCP

*Prunus salicina* Lindl. (Japanese plum, J)	Fresh fruit	Hot	EtOH	JFHE
			PG	JFHP
		Cold	EtOH	JFCE
			PG	JFCP
	Leaf	Hot	EtOH	JLHE
			PG	JLHP
		Cold	EtOH	JLCE
			PG	JLCP

*Prunus cerasifera* Ehrh. (cherry plum, C)	Fresh fruit	Hot	EtOH	CFHE
			PG	CFHP
		Cold	EtOH	CFCE
			PG	CFCP
	Leaf	Hot	EtOH	CLHE
			PG	CLHP
		Cold	EtOH	CLCE
			PG	CLCP

Abbreviations: EtOH = 95% ethanol, PG = propylene glycol.

**Table 2:** Yield percentages of extracts from plum leaves and fresh fruits in both hot and cold preparation processes extracted with ethanol and propylene glycol.

**Plum species**	**Code**	**Yield (%)**	**Code**	**Yield (%)**
*Prunus domestica* L. (European plum, E)	EFHE	45.75	ELHE	9.15
	EFHP	100	ELHP	100
	EFCE	55.82	ELCE	6.27
	EFCP	100	ELCP	100

*Prunus salicina* Lindl. (Japanese plum, J)	JFHE	34.85	JLHE	8.18
	JFHP	100	JLHP	100
	JFCE	50.64	JLCE	8.23
	JFCP	100	JLCP	100

*Prunus cerasifera* Ehrh. (cherry plum, C)	CFHE	35.25	CLHE	5.52
	CFHP	100	CLHP	100
	CFCE	48.84	CLCE	6.82
	CFCP	100	CLCP	100

**Table 3:** TPC, TFC, and antioxidant activity (DPPH) assays of ethanolic crude extract and propylene glycol extract solutions of plum leaves and fresh fruits.

**Sample extract**	**TPC (mg GAE/g sample)**	**TFC (mg QE/g sample)**	**DPPH (IC**_**50**_ **, *μ*g/mL)**
EFHE	15.43 ± 2.11^e,f,g^	7.85 ± 0.89^d^	1060.00 ± 7.21^b,c,d^
EFHP	10.39 ± 1.37^g^	0.21 ± 0.02^d^	3093.67 ± 462.26^a^
EFCE	24.72 ± 3.45^d,e^	8.10 ± 0.22^d^	296.35 ± 10.85^f,g,h^
EFCP	12.97 ± 0.52^f,g^	0.15 ± 0.02^d^	488.60 ± 30.00^e,f,g^
JFHE	16.86 ± 2.25^e,f,g,^	3.35 ± 0.48^d^	734.53 ± 27.10^c,d,e,f^
JFHP	15.17 ± 0.66^e,f,g^	0.12 ± 0.02^d^	2900.33 ± 234.40^a^
JFCE	20.06 ± 1.69^e,f,g^	3.14 ± 0.39^d^	339.77 ± 46.90^f,g,h^
JFCP	12.58 ± 0.87^f,g^	0.20 ± 0.02^d^	823.60 ± 105.50^b,c,d,e^
CFHE	20.94 ± 2.63^e,f,g^	4.24 ± 0.16^d^	713.33 ± 27.01^d,e,f^
CFHP	13.45 ± 1.06^f,g^	0.39 ± 0.04^d^	1169.00 ± 109.49^b,c^
CFCE	24.75 ± 2.78^d,e^	5.10 ± 0.14^d^	164.83 ± 20.67^g,h^
CFCP	15.68 ± 1.36^e,f,g^	0.17 ± 0.02^d^	375.93 ± 30.75^f,g,h^
ELHE	106.12 ± 7.82^a,b^	98.01 ± 5.03^c^	32.18 ± 2.75^h^
ELHP	21.63 ± 1.49^e,f^	6.71 ± 0.20^d^	962.13 ± 47.78^b,c,d^
ELCE	108.69 ± 2.96^a^	105.10 ± 11.31^c^	30.04 ± 1.88^h^
ELCP	32.77 ± 2.06^d^	11.38 ± 0.67^d^	622.03 ± 53.38^d,e,f^
JLHE	87.92 ± 3.23^c^	98.15 ± 5.36^c^	38.95 ± 0.66^h^
JLHP	22.90 ± 2.03^d,e,f^	7.67 ± 0.17^d^	2675.33 ± 308.21^a^
JLCE	112.34 ± 9.08^a^	148.67 ± 17.75^b^	31.44 ± 4.16^h^
JLCP	25.00 ± 2.83^d,e^	6.62 ± 0.17^d^	1195.87 ± 79.87^b^
CLHE	92.39 ± 4.66^c^	138.62 ± 20.04^b^	31.43 ± 1.28^h^
CLHP	23.42 ± 1.05^d,e,f^	6.02 ± 0.85^d^	2722.67 ± 329.31^a^
CLCE	96.94 ± 6.73^b,c^	185.94 ± 23.35^a^	29.97 ± 1.27^h^
CLCP	21.43 ± 1.39^e,f,g^	5.30 ± 0.59^d^	722.50 ± 51.02^d,e,f^
Rutin	—	—	12.55 ± 1.07^h^
Trolox	—	—	4.70 ± 0.33^h^

*Note:* The data are presented as mean ± SD with triplicates, and different letters (a–h) in the same column indicate significant differences (*p* < 0.05) using Tukey's HSD test.

**Table 4:** Amounts of rutin in various ethanolic crude extracts of plum leaves and fresh fruits.

**Extract sample**	**Amount of rutin (% ** **w**/**w)**
EFCE	12.85 ± 0.08^c^
JFCE	3.39 ± 0.02^a^
CFCE	6.26 ± 0.01^b^
ELCE	43.82 ± 0.13^f^
JLCE	28.52 ± 0.00^d^
CLCE	37.39 ± 0.07^e^

*Note:* The data are presented as mean ± SD (*n* = 3). Groups labeled with different letters within a column are statistically significantly different at *p* < 0.05 (Tukey's HSD).

## Data Availability

The data that support the findings of this study are available from the corresponding author upon reasonable request.
